# Mapping smart technologies and nutritional strategies for monitoring cognitive resilience in military personnel under extreme operational conditions: a scoping review

**DOI:** 10.3389/fnut.2026.1719702

**Published:** 2026-03-25

**Authors:** Dinu Țurcanu, Rodica Siminiuc

**Affiliations:** Faculty of Food Technology, Technical University of Moldova, Chisinau, Moldova

**Keywords:** adaptive behavior, biomarker monitoring, field assessment, human performance optimization, neurocognitive performance, operational stress, wearable analytics

## Abstract

In extreme operational environments, cognitive performance is critical for mission safety and decision accuracy; however, it is vulnerable to sleep deprivation, energy deficits, and multiple stressors. This scoping review aimed to map and synthesize evidence on nutritional strategies and smart technologies designed to monitor and support cognitive resilience in military personnel. Following the Joanna Briggs Institute (JBI) methodology and the Preferred Reporting Items for Systematic Reviews and Meta-Analyses extension for Scoping Reviews (PRISMA-ScR) guideline, studies published between 2010 and 2024 were identified through Web of Science, PubMed, and eLibrary.ru databases. Eligible records included interventions addressing nutrition, physiological monitoring, and cognitive outcomes in extreme settings. Nearly 79 studies met the inclusion criteria. A majority of the studies focused on metabolic or physiological markers, while direct cognitive endpoints were underrepresented. Nutritional supplements (42%) and personalized diets (31%) were predominant, whereas operational rations were rarely tested. Caffeine was consistently associated with improvements in vigilance and reaction time but showed limited effects on sustained attention. Smart technologies were applied inconsistently, with ecological validation reported in only 25% of cases. Superior standardization in reporting and field validation are needed to enhance comparability. Integrating nutritional countermeasures with wearable analytics emerges as a promising approach to sustain behavioral resilience and cognitive readiness under operational stress.

**Systematic review registration:**

https://doi.org/10.1371/journal.pone.0327649, Protocol publication; https://doi.org/10.17605/OSF.IO/EC7MG, OSF registration.

## 1 Introduction

*Rationale*: In the context of contemporary conflicts and the increasing prevalence of operations conducted in extreme environments, ranging from the Arctic and the subtropical deserts to urban theaters oversaturated with information, maintaining the cognitive resilience of military personnel has become a strategic priority (1, 2). Unlike physical performance, which can be supported through well-established energy regimes, cognitive performance remains vulnerable to sleep deprivation, cumulative stress, energy deficits, and exposure to multiple stressors that impair attention, working memory, and decision-making accuracy (3–9). Research over the past decade confirms that the physiological response to operational stress, characterized by accelerated catabolism, elevated interleukin-6 (IL-6), hepcidin, and cortisol levels, directly affects executive cognitive processes, making decision-making accuracy increasingly dependent on adequate nutritional support (10–12).

Beyond their cognitive implications, these physiological responses exert direct and measurable effects on eating behavior and short-term weight regulation. High operational load, sleep fragmentation, sustained sympathetic activation, and elevated energy expenditure frequently suppress appetite and disrupt hunger–satiety signaling, leading to delayed or insufficient food intake (13–15). Sleep restriction further alters leptin–ghrelin dynamics, amplifying appetite dysregulation under stress (16, 17). These patterns contribute to transient energy deficits and short-term body mass reductions consistently documented during military operations (18, 19). These mechanisms closely parallel stress-induced disruptions in appetite and metabolic regulation described in civilian populations, reinforcing the relevance of military contexts as an intensified model for studying eating-behavior dynamics under strain (20). Importantly, such stress-driven alterations mirror mechanisms observed in broader weight-related health problems, including impaired self-regulation and compensatory reward-driven eating (21, 22). Accordingly, operational stress has been discussed in the literature as an intensified context in which eating- and weight-related dysregulation mechanisms can be observed, providing a relevant background for examining nutritional strategies in military settings (23, 24).

From a care-oriented perspective, nutritional strategies in military settings are increasingly discussed not only in relation to performance enhancement but also as components of functional support and preventive management (25). Within military and occupational health frameworks, these approaches are conceptualized as elements of functional care aimed at preserving operational readiness and decision-making capacity under sustained stress rather than treating clinical pathology per se. In this sense, nutritional management in military contexts aligns with contemporary nutrition-care paradigms focused on prevention, early risk identification, and continuous functional monitoring rather than the treatment of established disease (26, 27). Modern nutrition management increasingly relies on integrated, technology-supported approaches that combine dietary planning with continuous physiological monitoring and adaptive feedback, particularly in high-risk and resource-constrained operational environments (28–30). Nevertheless, classical nutritional interventions, including caffeine administration and selected dietary supplements, have shown limited and context-dependent benefits and are rarely associated with consistent improvements in cognitive outcomes under extreme operational conditions (31–36).

In line with these developments, the emergence of smart technologies—including biosensors, physiological monitoring platforms, and artificial intelligence (AI)-driven algorithms for fatigue and attentional-decline analysis—has enabled real-time monitoring of physiological stress and cognitive dynamics in field conditions (37–41). At present, however, research on digital monitoring technologies and nutritional strategies has largely evolved along parallel trajectories, reflecting differences in disciplinary focus and study objectives. As a result, the interaction between physiological signals and nutritional factors in supporting cognitive resilience under extreme operational conditions remains insufficiently explored. Importantly, this reflects a broader conceptual and methodological gap at the field level, rather than a limitation inherent to individual studies. Beyond their direct cognitive applications, several smart technologies provide operationally relevant indicators of nutritional adequacy and appetite regulation, including energy expenditure, sleep architecture, heart-rate variability, and cumulative physiological strain. Integrating such physiological signals with nutritional data may enable earlier identification of energy deficits and dysregulated intake patterns during sustained operations, thereby supporting the development of evidence-based countermeasures to enhance both physical and cognitive readiness in military personnel.

*Objectives*: This scoping review aims to map and analyze the scientific literature addressing nutritional strategies and smart technologies used to support and monitor cognitive resilience among military personnel operating in extreme environments. Specifically, this review examines the types of military populations investigated, the nutritional and technological approaches applied, and the operational contexts in which these strategies have been evaluated.

To address the objectives of this scoping review, the following research questions were formulated:

*RQ1*. What types of military populations have been investigated in studies examining nutritional strategies and smart technologies related to cognitive resilience under extreme operational conditions?

*RQ2*. What nutritional strategies and smart technological tools have been employed to support or monitor cognitive resilience and related cognitive or physiological outcomes in military personnel?

*RQ3*. Under which extreme operational environments (e.g., cold, heat, altitude, isolation, and sustained psychological stress) have these strategies been evaluated?

*RQ4*. What evidence gaps exist in the present literature regarding validation, applicability, and integration of nutritional strategies and smart technologies in operational military settings?

## 2 Materials and methods

### 2.1 Protocol and registration

This scoping review was designed and reported in accordance with the Joanna Briggs Institute (JBI) methodology and the PRISMA-ScR guideline (42–44). The review followed a pre-specified scoping review protocol focused on descriptive evidence mapping and data charting according to the Population–Concept–Context (PCC) framework, published in PLOS One^1^ (45) and registered in the Open Science Framework (OSF).^2^

### 2.2 Eligibility criteria

The time frame established in the initial protocol (2000–2025) was subsequently adjusted to 2010–2025, following the identification of a limited number of relevant publications before 2010 and the delayed emergence of smart technologies in military research. This adjustment aimed to ensure the relevance and comparability of the included evidence. The eligibility criteria and review questions were structured according to the PCC framework, in line with the previously published study protocol.

The population comprised military personnel, including recruits, active-duty members, special forces, and support staff, evaluated during training programs, real missions, or relevant simulations. The Concept covered nutritional interventions such as operational rations, personalized diets, dietary supplements, and hydration protocols, along with smart technologies including wearable biosensors, mobile applications, and AI-driven platforms designed to monitor or support cognitive resilience. The Context referred to extreme operational environments characterized by severe climates, prolonged physical exertion, sleep deprivation, limited nutritional resources, deployment in hostile regions, or exposure to multiple concurrent stressors. Studies published in English, French, Spanish, German, Romanian, and Russian were included if they employed quantitative, qualitative, or mixed methods and explicitly reported outcomes related to cognitive performance. Studies focusing solely on economic or agricultural aspects, those employing exclusively pharmacological interventions, or those analyzing civilian populations without direct relevance to military settings were excluded.

### 2.3 Information sources

The registered protocol outlined a multi-database search strategy including PubMed/MEDLINE, Web of Science, Scopus, and IEEE Xplore databases, along with grey literature sources (CyberLeninka, NATO, WHO, and DARPA reports). During implementation, the final selection was narrowed to PubMed/MEDLINE, Web of Science, and eLibrary.ru, as pilot queries in the other databases yielded a high volume of redundant records or entries with limited relevance to the review objectives. This adjustment aimed to enhance search precision and feasibility while minimizing thematic duplication across sources. Although the search window extended to 2025, no eligible 2025 studies met the inclusion criteria, and the final evidence base reflects publications up to 2024.

### 2.4 Search strategy

The complete electronic search strategies, including query syntax and database-specific adaptations, were predefined in the published protocol^3^ (45), and are reproduced verbatim in Supplementary File S9 to ensure reproducibility. In the final searches, filters aligned with the eligibility criteria were applied, restricting results to the 2010–2025 period and accepted languages.

### 2.5 Selection of sources of evidence

**Figure 1:**
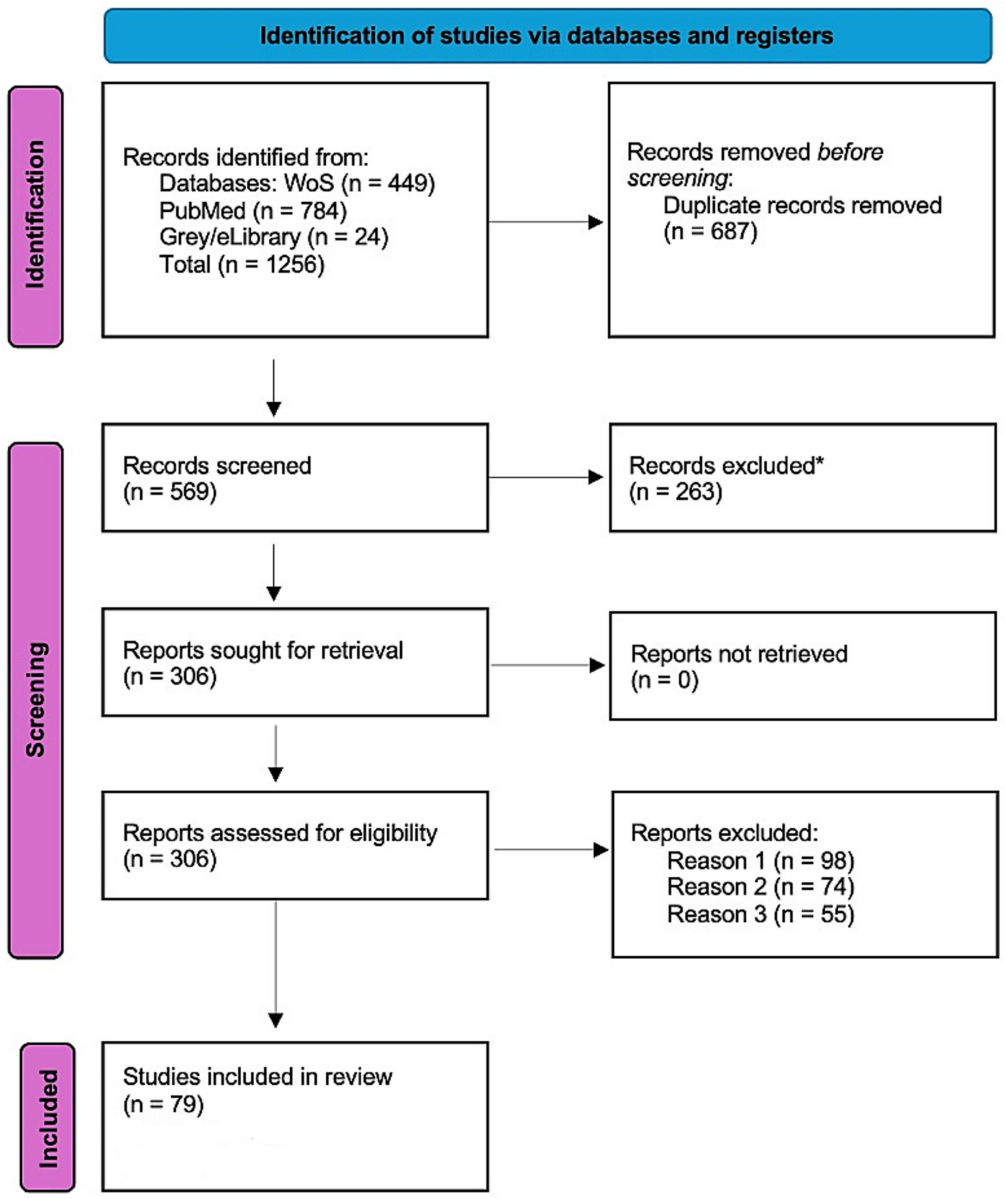
PRISMA-ScR flow diagram of the study selection process. Grey and regional literature includes publications from eLibrary.ru (*n* = 24). Records excluded during the screening stage (*n* = 263) were removed based on title and abstract review. Full-text excluded at the eligibility stage (*n* = 227) were grouped into three main categories: non-relevant population or context (*n* = 98), non-nutritional or out-of-scope interventions (*n* = 74), and non-cognitive outcomes (*n* = 55). No automation tools were used.

All identified records underwent a two-stage screening process. In the first stage, titles and abstracts were examined to apply the eligibility criteria defined in the protocol. In the second stage, preselected articles were reviewed in full to confirm inclusion. The process was conducted independently by two reviewers, with discrepancies resolved through discussion and consensus. The number of records screened, reasons for exclusion, and the final set of included studies are illustrated in the PRISMA-ScR flow diagram (Figure 1).

### 2.6 Data extraction process

For each included study, data were extracted using a standardized form developed in accordance with the PCC framework. The structure of the form was internally reviewed to ensure clarity and consistency prior to implementation. The form captured bibliographic information, methodological characteristics, and variables related to nutrition, monitoring technologies, and cognitive resilience, operationalized as described in Section 2.7. Data extraction was performed independently by two reviewers. In cases of incomplete reporting, the original sources were revisited; any discrepancies were discussed until consensus was reached.

### 2.7 Data elements

Data extraction followed the PCC framework defined in the published protocol (https://doi.org/10.1371/journal.pone.0327649.t001, *PLOS ONE*), focusing on three core dimensions. For the Population, data were collected on the categories of military personnel investigated and their operational roles. For the Concept, information was gathered on reported nutritional strategies and the use of smart technologies for monitoring and supporting cognitive resilience. For the Context, factors and challenges associated with deployment in extreme operational environments were documented.

In addition to the PCC elements, essential bibliographic information (title, authors, year, publication type, source, and digital identifier) and general methodological characteristics of the included studies were extracted. Furthermore, variables relevant to the outcome analysis were charted, including the biomarker types used to assess cognitive resilience, mentions of military nutrition standards, classification of reported effects (beneficial, neutral, or adverse), and their domains of impact (cognitive, psychological, physiological, and operational). To ensure consistency across studies, predefined harmonization rules were applied in cases of incomplete reporting. No assumptions beyond category harmonization were applied during data charting.

### 2.8 Critical appraisal

In accordance with the JBI guidance for scoping reviews, no formal critical appraisal of individual sources of evidence was performed, as the objective of this review was to map the extent, characteristics, and gaps of the existing literature rather than to assess intervention effectiveness.

### 2.9 Synthesis of results

Data were synthesized using a thematic and descriptive approach, in accordance with the PRISMA-ScR guidelines, combining narrative presentation with tabular and visual representations. Evidence was grouped and synthesized according to the predefined PCC framework, with additional aggregation by type of nutritional strategy, smart technology, and outcome domain. Descriptive numerical summaries (counts and frequencies) were used exclusively to support evidence mapping and gap identification; no inferential statistical analyses, effect size estimation, comparative effectiveness assessment, or meta-analysis were performed. Graphical analyses were performed using RAWGraphs (version 2.0; DensityDesign Research Lab, Politecnico di Milano, Italy) for conceptual visualizations (matrix plot and Sankey diagram) and GraphPad Prism (version 10.6; GraphPad Software, San Diego, CA, USA) for quantitative visualizations (stacked bar charts and heat maps). The consolidated dataset resulting from the charting process is presented in Supplementary Table S1 (Supplementary File S1).

## 3 Results

### 3.1 General characteristics of the included studies

**Figure 2:**
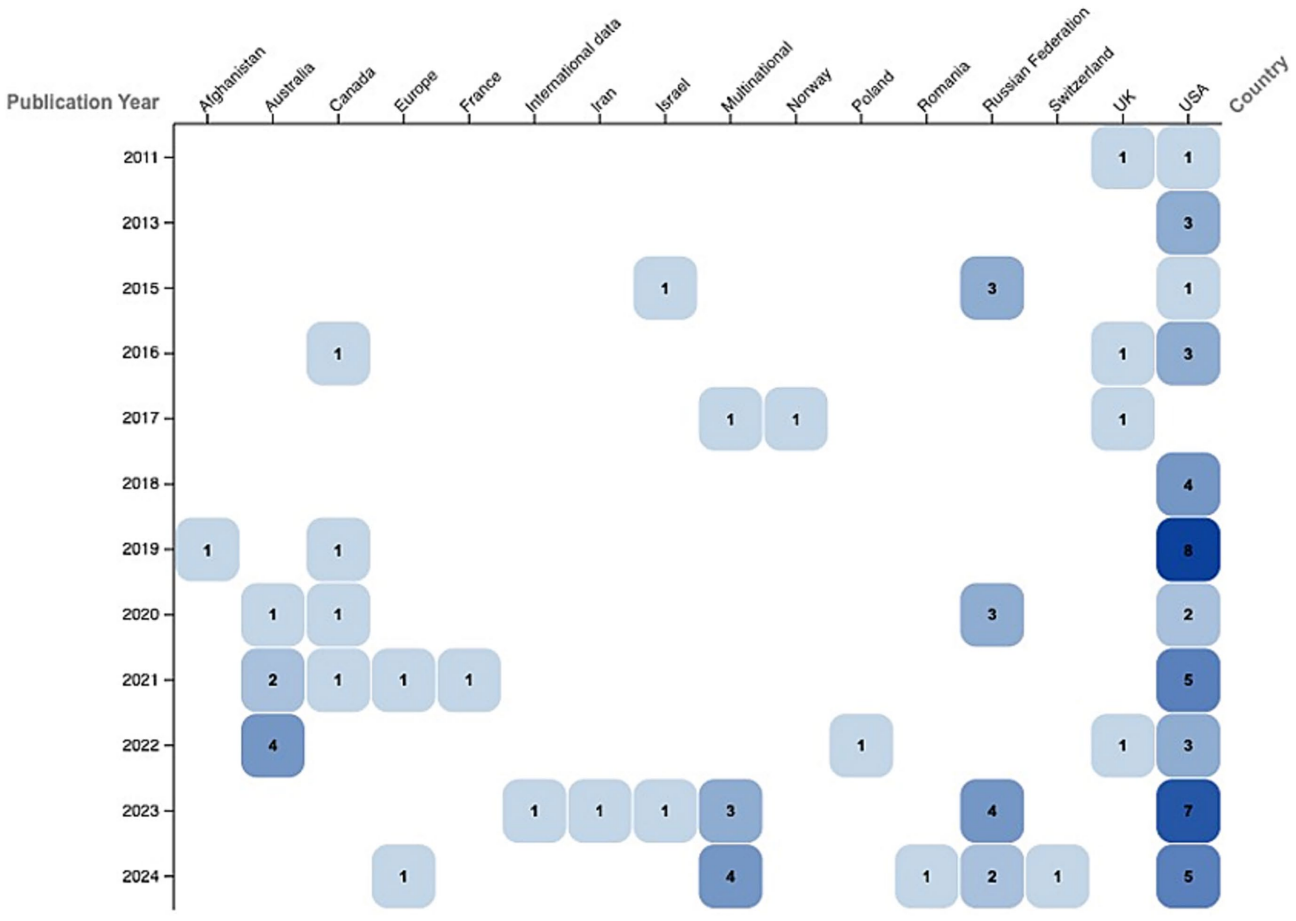
Distribution of scientific publications by country and year (2011–2024). Matrix plot showing publication counts by country and year. Cell size and color intensity reflect the number of publications; values represent absolute counts.

The results are synthesized descriptively as an evidence map aligned with the PCC framework, through thematic aggregation and visual mapping of the charted data, without quantitative pooling, inferential analysis, or meta-analysis. The final analysis comprised 79 publications (publication period: 2011–2024). The annual distribution was uneven, with a marked concentration of studies in the past 5 years. Annual peaks were observed in 2019 (*n* = 10), in 2023 (*n* = 17), and in 2024 (*n* = 14), while only 1–3 publications per year were recorded during the early stage (2011–2013) (Figure 2). Detailed study-level characteristics and charted data for each included source of evidence are provided in Supplementary Table S1.

### 3.2 Military populations and mission types

**Figure 3:**
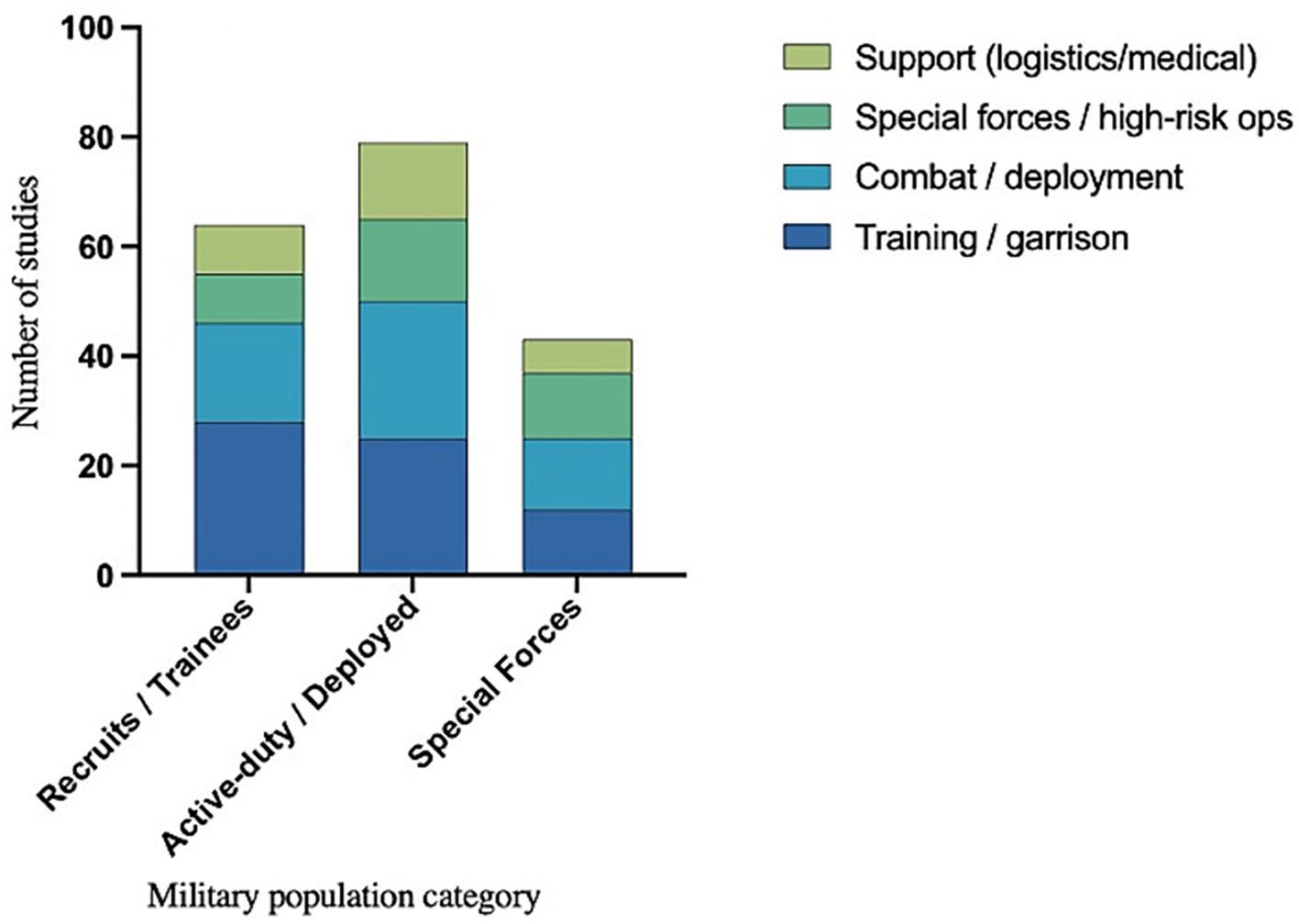
Distribution of military population categories by operational roles investigated in the included studies. Stacked bar chart illustrating the number of studies for each military personnel category (recruits/trainees, active/deployed personnel, and special forces) in relation to operational roles: training/garrison, combat/deployment, high-risk operations, and logistical/medical support. Peacekeeping roles were not identified (Supplementary Table S2).

The operational structure of the included studies was dominated by training and garrison activities, followed by combat or deployment missions, whereas high-risk operations and logistical/medical support roles were less represented. At the population level, recruits and trainees were primarily assessed in training contexts (28 out of 64), active-duty personnel showed an almost balanced distribution between training and combat (25 out of 79 each), and special forces demonstrated a slight predominance in combat/deployment settings (13 out of 43), with comparable proportions for training and high-risk missions (12 out of 43 each). Peacekeeping or stabilization roles were not reported (Figure 3).

### 3.3 Reported nutritional strategies across operational contexts

**Figure 4:**
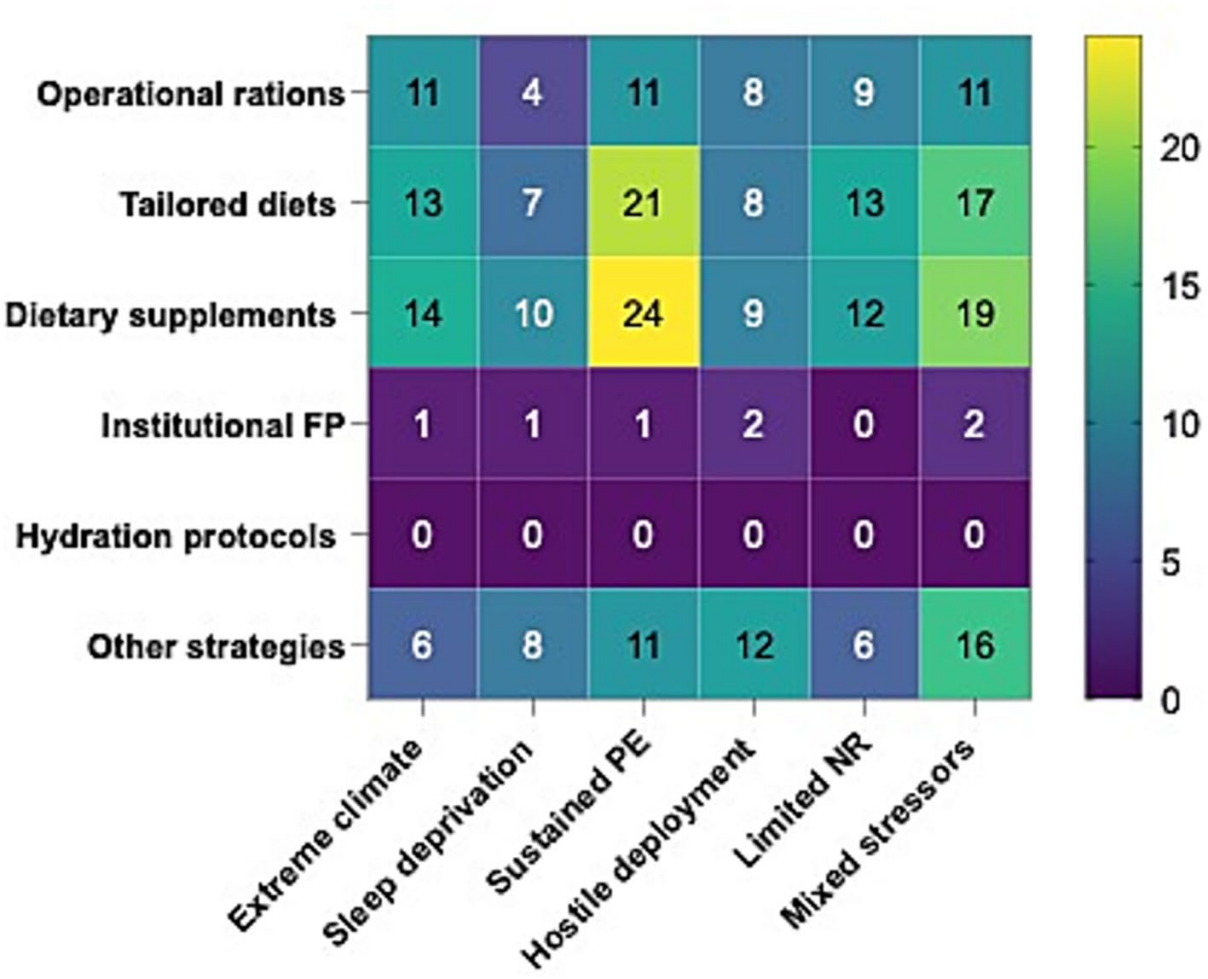
Distribution of nutritional strategies across extreme operational contexts. Heatmap showing the frequency of studies for each strategy–context combination (operational rations, personalized diets, supplements, institutional policies, hydration protocols, other strategies × extreme climate, sleep deprivation, sustained effort, deployment in conflict zones, limited resources, and combined stressors). Darker shades indicate higher concentration of evidence (Supplementary Table S3).

Across operational contexts, nutritional supplements and personalized diets were the most frequently reported strategies, consistently documented across all six scenarios. Deployment in conflict zones showed the highest frequency for both categories (supplements: 24 studies; personalized diets: 21 studies), followed by scenarios involving combined stressors (19 and 17 studies, respectively). In extreme climates, supplements (14 studies) and personalized diets (13 studies) were also common, suggesting the targeted use under intense physiological strain (Figure 4).

Operational rations showed moderate representation, particularly in extreme climates and conflict deployments (up to 11 studies). The “other strategies” category remained heterogeneous, reaching its peak in combined stressors (16 studies). Institutional nutrition policies were rare (no more than two studies per context), and hydration protocols were not reported, indicating a persistent gap in documentation.

### 3.4 Distribution of nutritional strategies, operational contexts, biomarkers, and effects on cognitive resilience

**Figure 5:**
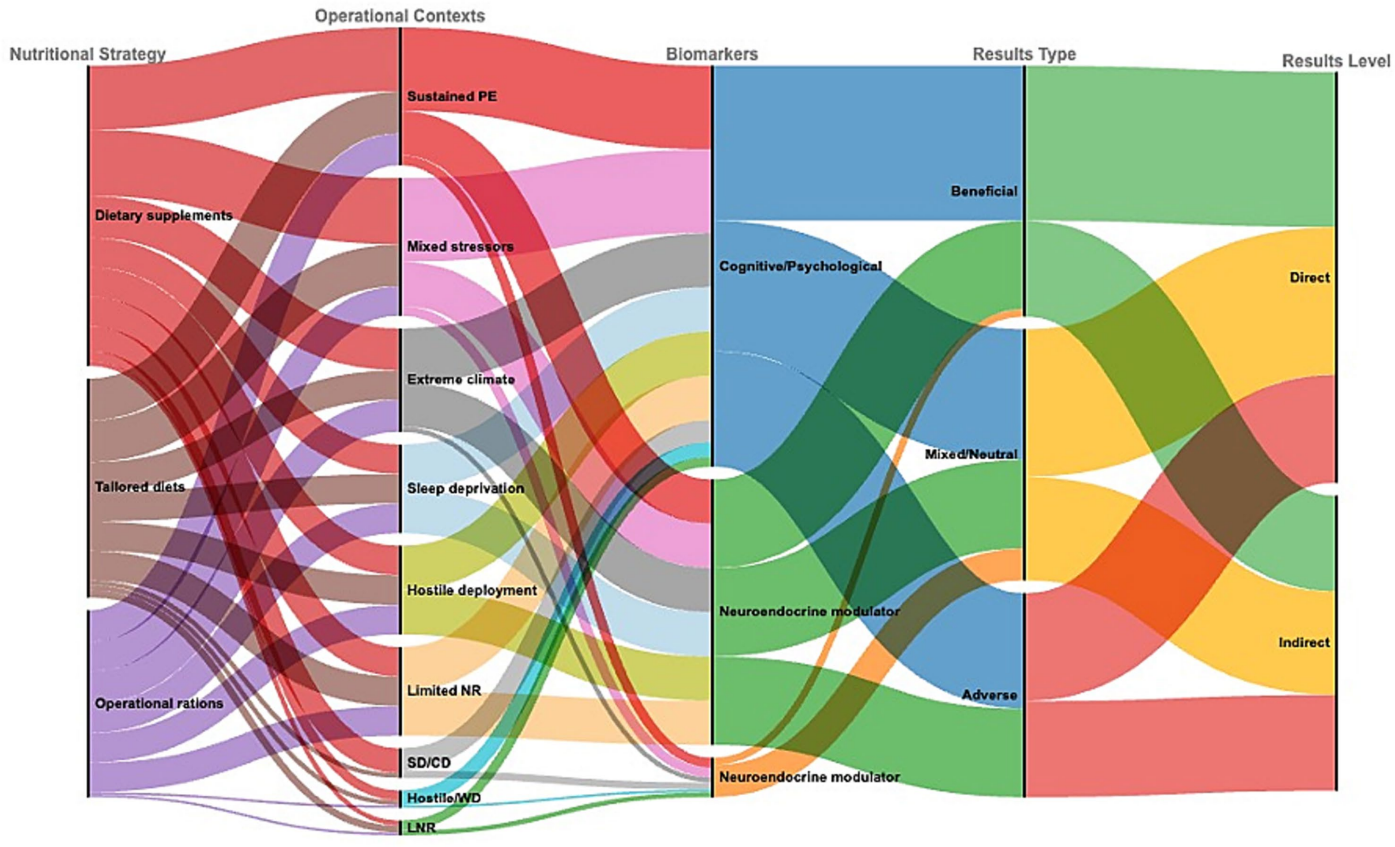
Interplay between nutritional strategies, operational contexts, biomarkers, and cognitive outcomes in extreme operational environments. Sankey diagram illustrating weighted links (by frequency) between strategies (operational rations, personalized diets, and supplements), operational contexts, biomarker categories (cognitive/psychological and neuroendocrine), and outcome types (beneficial, mixed/neutral, adverse; direct vs. indirect assessment). Arc width is proportional to the number of evidence units (*n* = 287) (Supplementary Table S4).

The dataset comprised 287 evidence units, each representing a unique combination of nutritional strategy, operational context, biomarker, effect type, and assessment level. Nutritional supplements accounted for 42.5% and were investigated in 45 out of 79 studies, followed by personalized diets (31.0%) and operational rations (26.4%) (Figure 5).

When examined at the level of evidence units, nutritional strategies, biomarkers, and reported effects showed a more granular distribution than at the study level, enabling the identification of co-occurrence patterns across strategies, physiological markers, and assessment modalities without re-aggregating results by operational context. Evaluations primarily focused on cognitive and psychological biomarkers (56.8%), while neuroendocrine modulators accounted for 43.2%. The distribution of reported effects was balanced between beneficial and mixed/neutral outcomes (35.5% each), with adverse effects observed in 28.9%. Most outcomes were derived from direct measurements (58.2%), while the remainder were assessed indirectly (41.8%).

### 3.5 Monitoring technologies and validation

Among the 79 included studies, 33 (42%) reported the use of *smart technologies* to monitor nutritional interventions, while 46 (58%) relied exclusively on conventional methods, such as dietary logs and standard biochemical assays. For the purpose of this scoping review, smart monitoring technologies were classified into two non-overlapping categories based on their mode of deployment and data acquisition. Wearable biosensors were defined as body-worn devices designed for continuous or near-continuous monitoring during routine activity or field operations (e.g., heart rate/heart rate viability (HR/HRV) sensors, actigraphy devices, core-temperature capsules, sweat, or biochemical sensors). In contrast, portable laboratory monitoring solutions were defined as transportable analytical systems requiring active measurement sessions or operator involvement, typically used intermittently in field or controlled settings (e.g., portable indirect calorimetry, biochemical analyzers, and mobile cognitive testing stations). Laboratory-based metabolic assessments, when deployed outside fixed clinical laboratory settings, were treated as a subset of portable laboratory monitoring solutions for both the classification and data charting purposes.

In practical applications, these technologies were deployed through body-worn sensors, portable analytical systems, and digital platforms for physiological and cognitive assessment. These systems monitored variables such as physical activity and energy expenditure, sleep duration and efficiency, autonomic load, core temperature, cognitive vigilance, and metabolic or physiological strain. This distinction was applied constantly during data charting to ensure comparability across studies. A structured summary of all smart technologies identified—detailing device type, function, monitored variables, and validation level—is provided in Supplementary Table S7.

**Figure 6:**
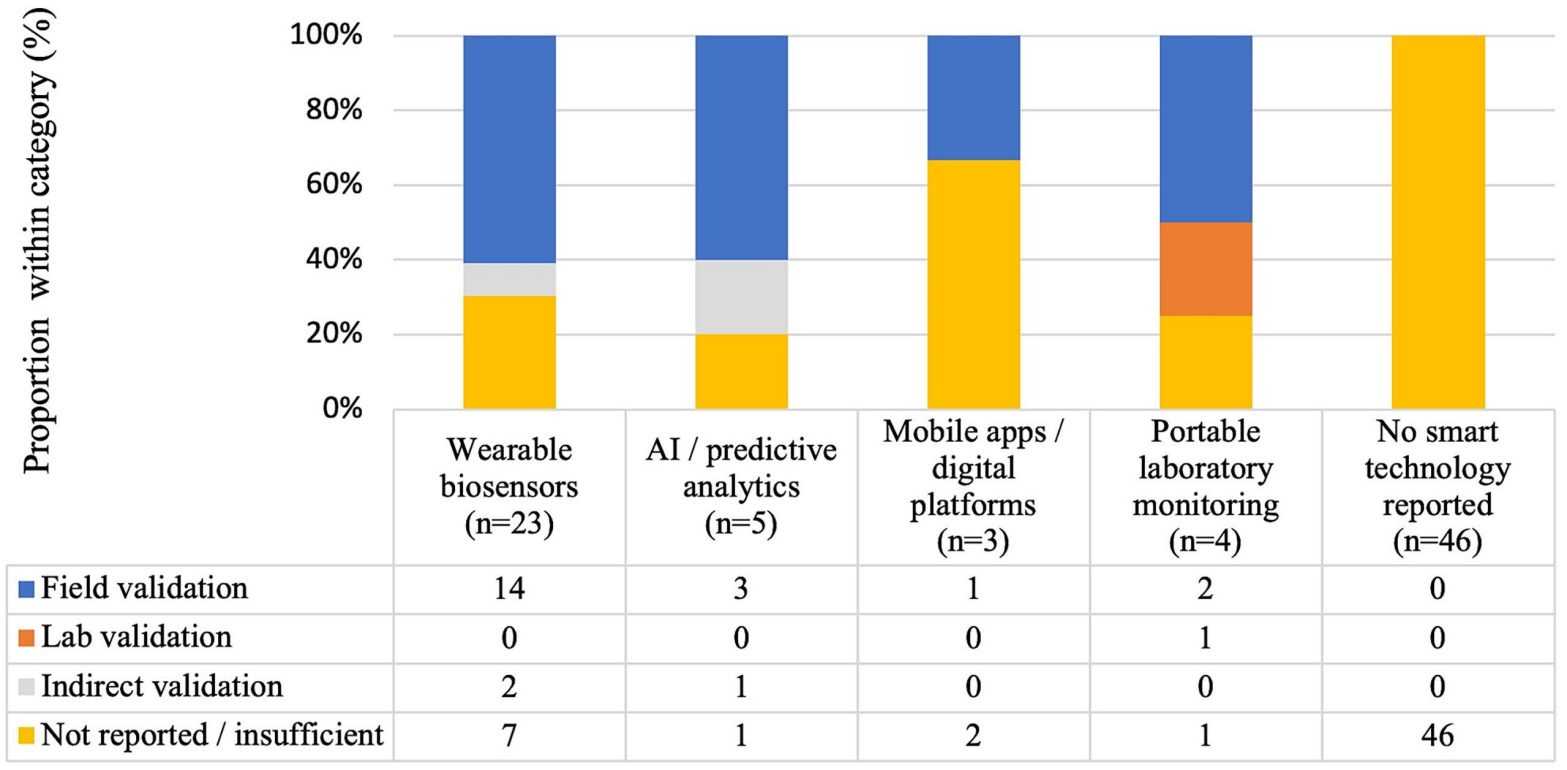
Reported validation level by monitoring-technology category. Stacked column chart showing validation levels (field, laboratory, indirect, and not reported) across wearable biosensors, predictive analytics, mobile apps, portable laboratory tools, and studies without smart technology. A single study may include multiple tools, so the number of technology-validation units exceeds the number of studies (Supplementary Table S5).

The most frequent category consisted of wearable biosensors (*n* = 23), followed by AI-based or predictive analytics systems (*n* = 5), mobile applications (*n* = 3), and portable laboratory monitoring solutions (*n* = 4). Several studies reported the concurrent use of multiple tools, illustrated in Figure 6.

A total of 81 technology–validation evidence units were identified. Field validation was reported in 20 out of 81 cases (24.7%), laboratory-only validation in 1 out of 81 (1.2%), indirect validation (via secondary correlations or literature reference) in 3 out of 81 (3.7%), and not reported/insufficient in 57 out of 81 (70.4%). Each evidence unit corresponded to a unique technology–study pairing, allowing multiple units to be derived from a single study when more than one monitoring tool was used. When restricting the analysis to smart technologies (*n* = 35 units), reflecting the use of multiple monitoring tools within individual studies, the distribution was as follows: field validation—20 out of 35 (57.1%), laboratory validation—1 out of 35 (2.9%), indirect validation—3 out of 35 (8.6%), and not reported/insufficient—11 out of 35 (31.4%). Across categories, wearable biosensors showed field validation in 14 out of 23 (60.9%), AI/predictive analytics in 3 out of 5 (60.0%), mobile apps in 1 out of 3 (33.3%), and portable laboratory monitoring tools in 2 out of 4 (50.0%) cases. Overall, the high proportion of not reported/insufficient validations highlights the need for standardized reporting criteria to ensure comparability and transparency in the field-ready technology assessments.

### 3.6 Military standards

References to military nutritional standards were frequent within the analyzed dataset; 41 studies (52%) cited at least one normative document. The U.S. Army Regulation (AR) 40–25: Nutrition and Menu Standards for Human Performance Optimization, together with the Military Dietary Reference Intakes (MDRIs), were predominant (*n* = 20) and used to define energy–protein targets and operational limits for caffeine intake. The second major group referred to operational ration standards, including Meal, Ready-to-Eat (MRE), First Strike Ration (FSR), Long-Range Patrol Ration (LRP), and Norwegian Arctic Ration (NAR), was reported in 13 studies, primarily for assessing field energy adequacy (Table 1).

**Table 1:** Military nutrition standards cited in the evidence base.

Standard/guideline	Classification	Pubs (n)	Sources
Military Dietary Reference Intakes (MDRI) or AR 40–25 (incl. Protein/energy targets, caffeine limits)	Nutrient-intake reference/menu standards	20	(46, 50, 55, 56, 64–75)
MRE, First-Strike Ration, Long-Range Patrol, Norwegian Arctic Ration, CAF allowances, etc.	Combat-ration standard	13	(11, 18, 47, 57, 68, 72–74, 76–81)
UK Military Dietary Reference Values (MDRV) + JSP 456	National nutrient reference	1	(82)
NATO Nutrition/Menu Standards (incl. 30-day CR limit, BW/BF monitoring)	International policy	1	(83)
Canadian Armed Forces (CAF) ration allowances/extreme-env. Guidelines	National policy	1	(47)
Russian Federation ration norms (Norma No. 1; 2007 Food Ration Norms)	National ration norm	2	(84, 85)
DoD caffeine-use guidelines (gum/capsules; operational dosage)	Supplement-control policy/performance countermeasure policy.	2	(31, 32)
USSOCOM supplement-control policies	Supplement-control policy/performance countermeasure policy.	2	(67, 80)
Army Body-Composition Program (ABCP/AR 600–9)	Regulatory document	3	(10, 86)
Total Force Fitness (TFF) readiness framework	Readiness framework	2	(87, 88)

The reported numbers refer to document mentions; the same study may cite multiple standards.

Non-U.S. standards were infrequently reported. The Military Dietary Reference Values (MDRV) and the JSP 456: Defence Catering Manual (United Kingdom), the North Atlantic Treaty Organization (NATO) menu/nutrition standards, and the Canadian Armed Forces Environmental Stress Guidelines for extreme environments were each mentioned in a single study, while the Russian Federation ration norms (Norma No. 1; Food Ration Norms, 2007) were cited in two sources. Additional thematic policies were cited intermittently, including the Department of Defense Caffeine-Use Guidelines (DoD) and the United States Special Operations Command (USSOCOM) supplement-use policies (each referenced in two publications), the Army Regulation 600–9: The Army Body Composition Program (AR 600–9) (three studies involving trainees), and the Total Force Fitness (TFF) framework (two studies).

## 4 Discussion

### 4.1 Central message and relevance to the ScR objective

The mapped evidence comprised predominantly experimental and quasi-experimental studies conducted in controlled, field-based, or mixed operational settings, complemented by a smaller number of observational and technology-focused studies, reflecting a heterogeneous but conceptually coherent evidence base aligned with the review objectives. This scoping review maps nutritional interventions and “smart” technologies aimed at supporting cognitive resilience in extreme operational environments, without estimating effects. The evidence map reveals a predominant focus on energetic and performance outcomes, with cognitive endpoints being underrepresented and digital technologies often incompletely reported or insufficiently validated. These patterns highlight the need for greater standardization and for field studies specifically targeting cognitive outcomes. Accordingly, the analysis provides a descriptive foundation for decision-makers, military planners, and researchers in operational nutrition seeking to integrate nutritional and technological approaches in support of cognitive resilience.

### 4.2 Interpretation of findings along the nutrition–technology–context axis

The distribution of nutritional strategies indicates a predominant focus on supplements and personalized diets, with fewer interventions tested directly through operational rations. In field studies and ecological models, repeated energy deficits characterize the norm rather than the exception, while inflammatory and iron metabolism responses intensify with physiological strain, potentially impairing cognitive performance—even when protein intake is elevated (5, 18). Evidence from Arctic training and climatic chamber experiments consistently showed energy intake below expenditure, accompanied by stress-induced appetite suppression, reduced voluntary energy intake, and rapid weight loss, which limited the capacity of interventions to preserve cognitive function if energy balance was not restored (46–48).

These operationally induced patterns mirror well-documented stress-driven alterations in civilian eating behavior, as described in the original studies under voluntary operational stress conditions, where physiological strain and sleep disruption disturb hunger–satiety signaling and impair metabolic regulation (49). This parallel reinforces the explanation for persistent energy imbalance, even in nutritionally optimized environments. At the ingredient level, caffeine remains the most consistent compound for sustaining vigilance under sleep deprivation, whereas other supplements demonstrate mixed or limited effects under operational conditions (31, 50). Even interventions with strong somatic effects, such as testosterone replacement in severe deficit, do not automatically translate into functional benefits, highlighting the need to include cognitive endpoints as primary outcomes in study design (10). Along the technological axis, the mapped literature reveals intermittent use of smart devices and frequent gaps in reporting or validation. Although wakefulness monitoring tools and portable cognitive tests can capture meaningful decrements during sustained operations, their sensitivity depends strongly on both the protocol design and the physiological data quality (39). Similarly, HRV remains as a potential indicator of acute stress and allostatic load; however, field generalization requires consistent standards regarding device type, sampling frequency, and artifact correction (37). Although these technologies are rarely applied with an explicit nutritional focus, the physiological markers they capture, such as energy expenditure trajectories, sleep fragmentation, autonomic load, and recovery dynamics, provide indirect yet operationally relevant cues regarding appetite suppression, inadequate intake, or emerging metabolic imbalance. When interpreted alongside structured nutritional data, such signals could enable real-time identification of individuals at risk for cumulative energy deficits or dysregulated eating patterns during extended operations.

Beyond cognitive monitoring, the mapping of the reviewed studies indicates that these technologies are also applied to capture physiological dimensions directly relevant to nutritional status and metabolic resilience. Wearable HR/HRV sensors and actigraphy-based energy-expenditure estimators provide continuous information on autonomic load, recovery capacity, and total metabolic demand-key determinants of individual energy requirements during training and deployment (51). Core-temperature capsules and thermal-strain monitors allow early identification of dehydration and heat-related metabolic stress, while portable indirect calorimetric systems quantify substrate use and shifts in oxidation pathways associated with malnutrition or high physiological strain. Molecular and biochemical biosensors further enable real-time detection of nutrient-related markers (e.g., lactate, electrolytes, hydration indicators), offering a dynamic picture of metabolic stress and nutritional adequacy in operational environments (52–54). These multilevel outputs demonstrate that digital technologies can support not only cognitive-performance surveillance but also integrated assessments of nutritional balance, fatigue accumulation, and physiological strain during sustained operations.

Overall, the absence of a clearly defined minimum dataset for sensors and of explicitly reported validation criteria limits inter-study comparability and undermines confidence in nutrition–physiology–cognition links derived from continuous monitoring. The operational context substantially modulates the interpretation of results. Heat, cold, altitude, sleep deprivation, and sustained physical load act synergistically, thereby exacerbating energy deficits, amplifying inflammation, and potentially altering the metabolic substrates required for cognitive performance. Under such conditions, even interventions with demonstrated laboratory efficacy may show attenuated or context-dependent effects (55–57). From this perspective, the interpretation of evidence should prioritize studies that simultaneously integrate energy balance control, direct cognitive measures, and standardized physiological stress monitoring in ecologically valid settings, as only such evidence can realistically estimate the contribution of nutritional interventions and digital technologies to cognitive resilience during operations. From a behavioral modeling perspective, operational stress adaptation can be conceptualized as a dynamic interaction between physiological strain, cognitive control, and adaptive behavior. Integration of behavioral metrics with nutritional and technological indicators may present a more comprehensive understanding of how individuals sustain decision-making stability and performance consistency under cumulative stress exposure.

Taken together, these findings delineate a coherent nutrition–technology–cognition pathway: nutritional interventions determine energy availability and metabolic stability; wearable sensors capture the resulting physiological strain, including autonomic load, sleep efficiency, thermoregulatory stress, and metabolic imbalance, while cognitive tests register the functional impact on attention, vigilance, and executive control (58–60). Sensor-derived markers therefore act as mediators linking nutritional status to cognitive performance. Elevated strain or cumulative sleep disruption detected through HRV or actigraphy frequently precedes decrements in reaction time or inhibitory control, clarifying why cognitive resilience deteriorates when energy balance is not restored.

### 4.3 Integrative interpretation within the P–C–C framework

The analysis reveals several limitations that may affect the generalizability and consistency of the conclusions. The mapped evidence base predominantly reflects training settings and physiological outcomes, as these contexts are more feasible and ethically less constrained than active operations. Consequently, the external validity for naval and air forces and missions with restricted nutritional resources remains limited. From a nutritional perspective, the persistent energy deficit documented in field conditions (46, 47) partially explains why supplements and personalized diets demonstrate stronger effects on metabolic markers than on primary cognitive endpoints, whereas caffeine remains the most consistently supported countermeasure for maintaining vigilance under sleep deprivation (31). Incorporating these mechanisms into future operational studies would improve the alignment between nutritional interventions, cognitive endpoints, and physiological monitoring. On the technological axis, the utility of HRV, actigraphy, and portable cognitive tests depends on ecological validation and standardized reporting (37, 61); otherwise, estimates risk introducing measurement bias. Overall, interpretation through the P–C–C lens highlights a gap between what is easily measurable (energy expenditure, body composition, and tactical performance) and what holds operational relevance (attention and decision-making under stress), underscoring the need to reposition cognitive outcomes as primary endpoints and to test interventions directly within operational rations and real-world theaters.

### 4.4 Gaps identified within the PCC framework

This evidence mapping (*N* = 79) reveals a recent upward trend in scientific output (peak in 2023, *n* = 17), yet with a disproportionate concentration on energetic/metabolic outcomes and overall performance, to the detriment of directly measured cognitive results. Only 36 records explicitly reported “Cognitive/Psychological” outcomes, and the “Type of effect = Cognition” category was identified only in six cases. This profile indicates that, although military nutrition is well represented in terms of study volume, the nutrition → cognition link remains underexplored and, consequently, less supported by robust cognitive indicators such as attention, memory, reaction time, and decision accuracy.

From a population perspective, a substantial proportion of the records focused on *recruits/trainees* (*n* = 35). This structure of the evidence base suggests a limited transferability of findings to experienced personnel or deployed settings, in which cognitive, physiological, and psychological demands are more pronounced. Similarly, only 10 studies were labeled under *Operational rations*, indicating a small volume of interventions tested directly on field rations in real-world conditions. Regarding military nutritional standards, 41 studies cited at least one normative or guideline document, while the remaining records did not report standards consistently. Such unequal and inconsistent reporting limits cross-study comparability and reduces the ability to assess the alignment of results with normative frameworks, such as energy adequacy and micronutrient intake. Furthermore, effect categories were reported non-uniformly, with 22 entries classified as *unspecified*, highlighting the need for enhanced standardization in both outcome definition and methodological transparency.

Another critical aspect concerns the documentation of “smart” technologies. A substantial proportion of studies relied on conventional approaches without smart technologies; however, explicit reporting of the technology absence or device characteristics was rare, with most entries lacking detailed documentation for this variable. From a scoping review perspective, this absence of explicit reporting denotes a gap either in documentation or in the actual use of wearable devices, sensors, and digital platforms for the continuous monitoring of cognitive status in operational environments. Considering the overarching objective of sustaining cognitive resilience under extreme conditions, the integration of such technologies remains an evident yet underexploited opportunity within the existing evidence base (Supplementary Tables S6a,b). Given the strong interaction between physiological strain, appetite suppression, and energy imbalance in extreme environments, a systematic integration of wearable-derived markers (energy expenditure, sleep, recovery, and autonomic load) may potentially enhance the ability to detect early nutritional inadequacy or stress-induced appetite suppression and the dysregulation of eating behavior, as suggested by the patterns observed across the mapped evidence. The absence of such integrated monitoring emerges from the mapped literature as a critical gap that limits real-time nutritional decision-making during operations.

### 4.5 Applied and operational implications

The evidence mapping indicates that cognitive outcomes are underrepresented as primary endpoints; when assessed, they are typically measured using validated batteries that assess attention, memory, reaction time, and decision-making accuracy. Such recalibration has been proposed for implementation in parallel with physiological monitoring, to establish mechanistic links between nutritional interventions and cognitive performance. To ensure external validity, an intervention assessment has predominantly moved from training settings toward real operational environments (heat, cold, and altitude), through field trials and observational cohorts involving deployed personnel rather than recruits or trainees. Inter-study comparability is currently limited by heterogeneous reporting practices, inconsistent alignment with the Military Nutrition Standards, and non-uniform classification of effect types, including frequent use of “Unspecified” labels. On the technological axis, the literature increasingly emphasizes the importance of the systematic integration of digital technologies [wearables, actigraphy, HRV, and computerized Psychomotor Vigilance Test (PVT)] under a minimum reporting protocol (sensor type, sampling frequency, variables, artifact control, accuracy, and data protection), enabling longitudinal monitoring and modeling of nutrition–cognitive resilience. The data also support an explicit focus on operational rations, where optimizing their composition and testing cognitive effects in realistic field scenarios, including non-inferiority trials versus standard regimens, represent immediate and actionable priorities for military doctrine and logistics.

### 4.6 Limitations

A formal appraisal of study quality was not conducted, as this step is generally considered outside the scope of a scoping review. The results rely exclusively on information reported in the primary sources; incomplete fields or entries marked as “Unspecified” may introduce classification bias and influence interpretation.

Terminological heterogeneity and inconsistent standardization of variables, particularly regarding the “type of effect” labeling and reporting against the Military Nutrition Standards, reduce inter-study comparability. Additionally, the overrepresentation of recruits and the limited number of studies conducted in extreme operational environments constrain the generalizability of findings to active or deployed personnel. Incomplete reporting of “smart” technologies prevents an accurate estimation of the degree of digital integration in field research, while the small number of interventions tested directly with operational rations weakens the evidence base for doctrine- and logistics-oriented recommendations. The search strategy covered major indexed databases (Web of Science, PubMed, and eLibrary.ru) and one grey literature source (CyberLeninka); however, the exclusion of additional specialized databases (e.g., Scopus, IEEE Xplore) may have reduced comprehensiveness. The analyzed period (2010–2025), adjusted from the initial protocol (2000–2025), may have excluded earlier emerging contributions. These constraints should be considered when interpreting the findings and may inform future methodological work aimed at improving standardization, contextual diversity, and technological integration.

### 4.7 Future directions—expanded and aligned with the objective

Strengthening the evidence base requires redefining cognitive performance as a primary outcome in randomized trials, evaluated alongside physiological markers and reported in accordance with CONSORT standards (62, 63). Future trials should be designed to integrate nutritional interventions, smart monitoring technologies, and validated cognitive outcomes within a unified experimental framework, as this intersection remains underrepresented in the contemporary literature. For ecological validity, pragmatic studies conducted under real operational conditions, including heat, cold, and altitude exposure, can provide a more realistic understanding of nutrition–cognition interactions and adaptive processes. Standardization remains a cross-cutting priority: adopting a shared taxonomy for the “type of effect,” documenting compliance with the Military Nutrition Standards, and establishing a “minimum dataset” for wearable technologies and mobile applications would substantially enhance inter-study comparability. The evaluation of operational rations in real-world settings, including non-inferiority trials, is essential for practical applicability. Finally, defining a “core outcome set” for cognitive resilience, together with implementation research on the feasibility and cost-effectiveness of digital solutions, may accelerate the translation of evidence into operational practice.

## 5 Conclusion

This scoping review highlights a marked imbalance between the extensive physiological evidence base and the limited number of studies directly assessing cognition, further compounded by inconsistent reporting, limited integration of digital technologies, and a predominant focus on training populations. In line with the study objective and PCC framework, this study conclude that operational relevance can be strengthened through:

(i) repositioning cognitive performance as a primary outcome in studies that simultaneously integrate nutritional interventions and smart monitoring technologies, measured with validated and domain-appropriate instruments;
(ii) evaluating interventions under real-world field conditions among deployed personnel, using operational rations; and
(iii) adopting shared reporting standards that include specifications for monitoring devices and nutritional compliance.

This direction transforms the present evidence map into an actionable decision-support tool, bridging the gap between research outputs and the real demands of operational missions. Beyond methodological refinement, future research integrating behavioral indicators may enhance the understanding of how nutrition and technology jointly sustain cognitive readiness and adaptive performance under extreme stress.

## Data Availability

The original contributions presented in the study are included in the article/ Supplementary material; further inquiries can be directed to the corresponding author.

## Footnotes

1
https://doi.org/10.1371/journal.pone.0327649

2
https://doi.org/10.17605/OSF.IO/EC7MG

3
https://doi.org/10.1371/journal.pone.0327649.s001
