# Supplementary material for: Mapping smart technologies and nutritional strategies for monitoring cognitive resilience in military personnel under extreme operational conditions: a scoping review
Source: Front Nutr. 2026 Mar 25;13:1719702. doi: 10.3389/fnut.2026.1719702 (PMC13056688; doi:10.3389/fnut.2026.1719702)
Supplement: Supplementary file 3 [file Table_3.DOCX]

**Supplementary File S9. Search strings_Full electronic search strategies**

**Table A1. Summary of electronic search strategies**

| 1. | **Initial Integrated Search (4 concepts – 0 hits)** |
| --- | --- |
|  | *Search strategy note:*  *The initial 4-concept query retrieved no references. We therefore adopted a revised 3-concept integrated search (row 2) and two complementary thematic searches (row 3). Results from all queries were pooled, deduplicated and screened together.*  ("Military Nutrition"[MeSH] OR "Combat Rations" OR "Dietary Supplements" OR "Operational Rations") AND ("Cognitive Resilience" OR "Cognitive Performance"[MeSH] OR "Mental Resilience" OR "Decision-Making") AND ("Military Personnel"[MeSH] OR "Armed Forces" OR "Soldiers" OR "Military Operations") AND ("Wearable Sensors" OR "Smart Devices" OR "Biomonitoring"). |
| 2. | **Revised Integrated Search (3 concepts – used for screening)** |
|  | ("military nutrition" OR "combat rations" OR "dietary supplements") AND ("cognitive resilience" OR "cognitive performance" OR "decision-making") AND ("soldiers" OR "military personnel" OR "armed forces") |
| **3.** | **Thematic Sub-Searches (to maximise sensitivity)** |
| 3.1. | **Search on Cognitive Resilience in Military Context** |
|  | ("Cognitive Resilience" OR "Cognitive Performance" OR "Mental Resilience") AND ("Military Personnel" OR "Armed Forces" OR "Soldiers") |
| 3.2. | **Search on Smart Technologies in Military Context** |
|  | ("Smart Technology" OR "Wearable Devices" OR "Biomonitoring" OR "Artificial Intelligence") AND ("Military Applications" OR "Defense Technology" OR "Military Operations") |

**Search Filters Applied**

- **Language:** English, French, Spanish, German, Romanian, and Russian.
- **Publication Date:** 2010-2025
- **Document Type:** No restriction applied (all study types included at this stage)
- **Access Type:** No access restrictions were applied. Articles behind paywalls will be accessed via institutional library resources or interlibrary loan services where needed.

**Table A 2. Scientific databases, grey literature, and governmental/military sources used in the search strategy**

| **Category** | **Database / Platform** | **Provider / Organization** | **Access Link** |
| --- | --- | --- | --- |
| **Scientific Databases** | **PubMed/ MEDLINE** | National Center for Biotechnology Information (NCBI) | [pubmed.ncbi.nlm.nih.gov](https://pubmed.ncbi.nlm.nih.gov/) |
|  | **Web of Science** | Clarivate Analytics | [www.webofscience.com](https://www.webofscience.com/) |
|  | **Scopus** | Elsevier | [www.scopus.com](https://www.scopus.com/) |
|  | **IEEE Xplore** | Institute of Electrical and Electronics Engineers (IEEE) | [ieeexplore.ieee.org](https://ieeexplore.ieee.org/) |
| **Grey Literature** | **CyberLeninka** | Independent academic repository | [cyberleninka.ru](https://cyberleninka.ru/) |
|  | **eLIBRARY.ru** | Russian Science Citation Index | [elibrary.ru](https://www.elibrary.ru/) |
| **Governmental / Military Sources** | **NATO** (North Atlantic Treaty Organization) | NATO Science & Technology | [www.nato.int](https://www.nato.int/) |
|  | **WHO** (World Health Organization) | World Health Organization (WHO) | [www.who.int](https://www.who.int/) |
|  | **DARPA** (Defense Advanced Research Projects Agency) | U.S. Department of Defense | [www.darpa.mil](https://www.darpa.mil/) |

**Note:** Although Scopus and IEEE Xplore were included in the initial search protocol, the final searches were conducted in PubMed/MEDLINE, Web of Science, and eLibrary.ru, as detailed in the Methods section.
